# Lessons learned from the investigation of a COVID-19 cluster in Creil, France: effectiveness of targeting symptomatic cases and conducting contact tracing around them

**DOI:** 10.1186/s12879-021-06166-9

**Published:** 2021-05-19

**Authors:** Franck de Laval, Anaïs Grosset-Janin, François Delon, Alexandre Allonneau, Christelle Tong, Flavie Letois, Anne Couderc, Marc-Antoine Sanchez, César Destanque, Fabrice Biot, Françoise Raynaud, Christine Bigaillon, Olivier Ferraris, Etienne Simon-Loriere, Vincent Enouf, Dinaherisoa Andriamanantena, Vincent Pommier de Santi, Emilie Javelle, Audrey Mérens

**Affiliations:** 1 SSA (French Military Health Service), CESPA (French Armed Forces Center for Epidemiology and Public Health), Epidemiological Surveillance and Investigations Unit, BdD Marseille-Aubagne, 111 Avenue de la Corse, Marseille, 13568 France; 2 Aix-Marseille University, INSERM, IRD, SESSTIM (Economic and Social Sciences, Health Systems, and Medical Informatics), Marseille, France; 3 SSA (French Military Health Service), 1st Armed Forces Medical Center, Paris, France; 4 SSA (French Military Health Service), Armed Forces Biomedical Research Institute, Brétigny-sur-Orge, France; 5 Direction Générale de l’Armement, Maîtrise NRBC, Vert-le-Petit, France; 6 SSA (French Military Health Service), Bégin Military Teaching Hospital, Saint-Mandé, France; 7 G5 Evolutionary genomics of RNA viruses, Institut Pasteur, Paris, France; 8 National Reference Center for Respiratory Viruses, Molecular Genetics of RNA Viruses, Institut Pasteur, CNRS—UMR 3569, University of Paris, Paris, France; 9 Mutualized Platform of Microbiology, Pasteur International Bioresources Network, Institut Pasteur, Paris, France; 10 Aix-Marseille University, IRD, AP-HM, SSA (French Military Health Service), VITROME, Marseille, France; 11 SSA (French Military Health Service), Laveran Military Teaching Hospital, Marseille, France

**Keywords:** SARS-CoV-2, COVID-19, Cluster, RT-PCR, Asymptomatic

## Abstract

**Background:**

This study presents the methods and results of the investigation into a SARS-CoV-2 outbreak in a professional community. Due to the limited testing capacity available in France at the time, we elaborated a testing strategy according to pre-test probability.

**Methods:**

The investigation design combined active case finding and contact tracing around each confirmed case with testing of at-risk contact persons who had any evocative symptoms (*n* = 88). One month later, we performed serology testing to test and screen symptomatic and asymptomatic cases again (*n* = 79).

**Results:**

Twenty-four patients were confirmed (14 with RT-PCR and 10 with serology). The attack rate was 29% (24/83). Median age was 40 (24 to 59), and the sex ratio was 15/12. Only three cases were asymptomatic (= no symptoms at all, 13%, 95% CI, 3–32). Nineteen symptomatic cases (79%, 95% CI, 63–95) presented a respiratory infection, two of which were severe. All the RT-PCR confirmed cases acquired protective antibodies.

Median incubation was 4 days (from 1 to 13 days), and the median serial interval was 3 days (0 to 15). We identified pre-symptomatic transmission in 40% of this cluster, but no transmission from asymptomatic to symptomatic cases.

**Conclusion:**

We report the effective use of targeted testing according to pre-test probability, specifically prioritizing symptomatic COVID-19 diagnosis and contact tracing. The asymptomatic rate raises questions about the real role of asymptomatic infected people in transmission. Conversely, pre-symptomatic contamination occurred frequently in this cluster, highlighting the need to identify, test, and quarantine asymptomatic at-risk contact persons (= contact tracing). The local lockdown imposed helped reduce transmission during the investigation period.

## Introduction/background

SARS-CoV-2 is a novel coronavirus that emerged in 2019 in Wuhan (China) and spread rapidly to become a pandemic via airborne, droplet, and hand-to-hand transmission [1]. It is responsible for an upper and/or lower acute respiratory infection of infected cases, ranging from mild and/or nonspecific to severe coronavirus disease called COVID-19 [1–3]. The first European cases were detected in France in January 2020 [4]. The French outbreak response included a three-stage strategy, with the first two stages being the early detection and isolation of every imported and then locally-acquired case, with rigorous contact tracing to contain local transmission. When the extent of the epidemic made the first two stages unmanageable, we moved on to the third stage, which focused strictly on the management of the most severe cases in healthcare facilities with an increase in hospital resources. The third stage also included a national lockdown starting on March 17, 2020 to slow down the reproduction rate and the geographic progression of the virus [5, 6]. After a remission stage, incidence has been significantly increasing in France and in other European countries, especially with the spread of SARS-CoV-2 variants [7]. Knowledge on the durability of the specific immune response following infection with coronavirus as SARS-CoV-2 currently remains uncertain [8, 9]. Furthermore, the possible ability of variants to escape post-infection or vaccine-induced immunity is concerning [10]. That is why we should not rely solely on the expectation of reaching a supposed minimal natural or vaccinal herd immunity to control the spread of the virus. Indeed, in a non- or partially immune population, a single infected person could restart an epidemic. In that hypothesis, the virus is at high risk for local re-circulation or re-importation if the same comprehensive, strict strategy is not implemented worldwide, and this could last several months or years [11]. Thus, management of emergences, clusters, outbreaks and contact tracing around them will still be part of the strategy. Feedback from recent experiences can improve the future response to the re-circulation of SARS-CoV-2.

In February 2020, a local COVID-19 cluster occurred in the French department of Oise [12]. It was the first cluster in France not linked to an imported case. Nested within this cluster, the transmission chain spread into a military support facility located at the air base in the city of Creil (MSFAC). We present here the investigation and management of this COVID-19 cluster. The main objective is to show that prioritizing symptomatic COVID-19 diagnosis and contact tracing led to successful containment.

## Methods

In accordance with French national recommendations in February 2020, every patient with COVID-19 symptoms *and* a known exposure risk required a SARS-CoV-2 RT-PCR test on a naso-pharyngeal sample. On February 25, 2020, the French National Reference Center (CNR) for respiratory infections confirmed Case 7 of the cluster described here. This case, which had a severe clinical presentation, was immediately notified to the French health authorities. The patient worked in the MSFAC. Immediately, an investigation was performed to identify backward and forward transmission chains around this case, manage the cases that were identified, and take countermeasures to contain viral transmission.

The investigation design combined active case finding and contact tracing around each case: 1/ Anyone from MSFAC with any symptoms was asked to report to the military health facility to undergo a RT-PCR test; 2/ Contact persons with any evocative symptoms had a RT-PCR test. Once the perimeter of the cluster was well defined (as of March 1), we considered the entire MSFAC staff as contacts and extended the RT-PCR indication to any symptomatic patients from MSFAC regardless of their contact history.

Confirmed cases were patients with positive RT-PCR test results and/or positive serology. Contact persons were persons with moderate to high risk of exposure to SARS-CoV-2, i.e. at-risk contact with a confirmed case according to French national recommendations (same household, room, team, etc., or about 15 min face to face < 1 m). Suspected cases were excluded after one or two negative RT-PCR tests (when the first sample was collected within 48 h of symptom onset or in case of real clinical suspicion).

A field sampling unit was set up in the military health facility to support the epidemiological investigation. It respected technical, biosafety, and biocleaning conditions and could perform 16–20 medical evaluations and test samples a day. Two swabs (nasopharyngeal and oropharyngeal) per case were sampled (Sigma Virocult, Medical Wire Instrument, Corsham) and pooled, stored at + 4 °C, and sent under biosafety conditions to the microbiology facility near Paris. We used the automated EZ1 XL (Qiagen France SAS, Courtaboeuf) for RNA extraction, following the manufacturers’ instructions, and a LightCycler 480 System (Roche Diagnostics, Meylan, France) for SARS-CoV-2 RT-PCR. Primers and probe sequences corresponded to the RdRp-IP2 and RdRp-IP4 assay designed at the CNR, and they provided positive SARS-CoV-2 control. For each specimen, the quality of the initial sampling, quality of RNA extraction, and the absence of PCR inhibitors were checked by two other PCRs, using a cellular control (CELL control R-gene, Argene, Biomérieux, France) and an internal control (RICO Extra-R-gene, Argene, Biomérieux, France). We sent positive samples to the CNR for sequencing of viral genomes to contribute to molecular epidemiological studies and, in particular, to investigate the link with a community cluster in Oise. We also collected the results of RT-PCR tests when they were performed in other laboratories.

As soon as serology assays were available, 1 month after this outbreak, we offered a serological test to all MSFAC staff to search for recent contact with SARS-CoV-2 and asymptomatic SARS-CoV-2-infected patients. Sera were tested by SARS-CoV-2 IgG and IgA assay (Euroimmun AG, Lübeck, Germany) on automated microtiter plate analyzers, Etimax (Diasorin SA, Antony, France) or Elispeed (Euroimmun). Positive sera for IgG or IgA with Euroimmun assay were controlled with Elecsys Anti-SARS-CoV-2 immunoassay (Roche Diagnostics, Meylan, France) on Cobas 6000. An in-house seroneutralization assay was performed [13]. If a discrepancy was found, a new serological test was proposed 2 weeks later.

Viral genome sequencing was attempted with a highly multiplexed PCR amplicon approach [14] using the ARTIC Network multiplex PCR primers set v1 (https://artic.network/ncov-2019), with modification as suggested in previous research [15]. Synthesized cDNA was used as a template, and amplicons were generated using two pooled primer mixtures for 35 rounds of amplification. Libraries were then prepared using the Nextera XT DNA Library Prep Kit (Illumina) and sequenced on an Illumina NextSeq500 (2 × 150 cycles).

Raw reads were trimmed using Trimmomatic v0.36 [16] to remove Illumina adaptors and low quality reads as well as primer sequences corresponding to the PCR amplicons. We performed iterative mapping against the reference genome Wuhan/Hu-1/2019 (NCBI Nucleotide – NC_045512, GenBank – MN908947) and then on the extracted consensus using the CLC Genomics Suite v5.1.0 (QIAGEN). We used SAMtools v1.3 to sort the aligned bam files and generate alignment statistics [17]. Aligned reads were manually inspected using Geneious prime v2020.1.2 (2020) (https://www.geneious.com/), and consensus sequences were generated using a minimum of 3X read-depth coverage to make a base call. No genomic deletions were detected in the genomes analyzed.

In addition, SARS-CoV-2 RT-PCR tests were performed on environmental swabs sampled from several common objects or surfaces inside the MSFAC building on March 1, before disinfection.

All confirmed cases were isolated for 14 days and medically monitored daily at home or at the hospital, according to symptom severity, individual risk of worsening illness, and proximity with persons in their household at risk for severe COVID-19 (vulnerable individuals). Contact tracing was performed remotely by phone. Cases were interviewed at length about symptoms and date of onset and about their activities and contacts in the 14 days prior to symptom onset, to determine the most probable source of the contamination, and in the last 48 h to identify at-risk contact persons. The interviewers used a standardized questionnaire.

After the investigation, strict epidemiological surveillance of COVID-19 was maintained to detect any new cases and respond rapidly. We used R software to calculate 95% confidence intervals.

## Results

### Case description

**Fig. 1:**
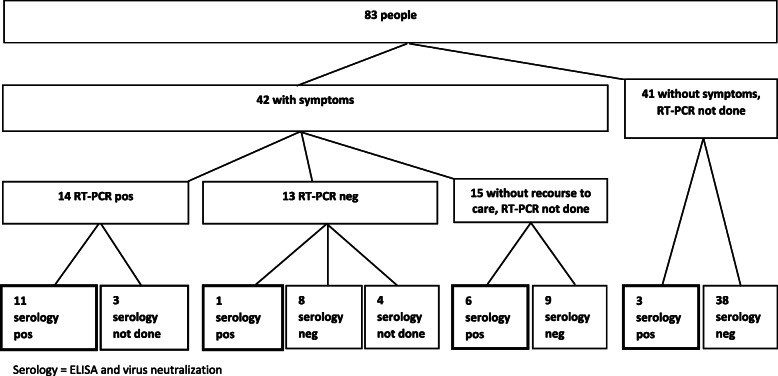
Flow chart of the outbreak investigation in MSFAC, Creil, February–March 2020 (*N* = 83)

From February 25 to March 4, 2020, 24 cases were confirmed: 14 with RT-PCR and 10 with serology (Fig. 1). Three had been totally asymptomatic (3/24, 13%, 95% CI, 3–32).

All RT-PCR confirmed cases (or their relatives, if necessary) and 323 contact persons, 119 of whom had real at-risk contacts, were interviewed. These 119 contacts were quarantined at home for 14 days and were supervised daily by phone. Sixty-one became symptomatic and required a RT-PCR test, three of which were positive. Among these three additional confirmed cases, two were from other facilities at the air base (Table 1).

**Table 1:** Clinical, biological, and epidemiological features of the 27 SARS-CoV-2 confirmed cases, Creil, February–March 2020

Clinic					Biology							Epidemiology				
					RT-PCR			Serology								
Case	Age	Symptoms	Date of symptoms	Symptoms duration (day)	Results interpretation	Ct IP2	Ct IP4	ELISA EuroImmun IgG/IgA	Roche	Titer of viro neutralisation	Results interpretation	Work place	Desk room number in MSFAC	Main contact with	Serial interval (day)	Incubation (day)
1	30–39	yes	03/02/20	NA	pos	37	34	ND	ND	ND	ND	City of Crépy-en-Valois	–	Index case	–	–
2	20–29	yes	05/02/20	3	pos	39	32	6.07/3.7	80.15	1/40	pos	MSFAC	A	Case 1	2	4
3	50–59	yes	05/02/20	5	ND	ND	ND	1.33/1.4	0.9	1/40	pos	MSFAC	H	Case 2	0	1
4	30–39	yes	07/02/20	20	pos	36	33	10.8/9.3	27.14	1/160	pos	MSFAC	A	Case 2	2	2–4
5	50–59	yes	08/02/20	24	pos	NA	NA	10.9/9.4	15.79	1/160	pos	MSFAC	B	Case 2	3	4–5
6	20–29	yes	08/02/20	NA	pos	36	34	ND	ND	ND	ND	MSFAC	A	Case 2	3	4–5
7	50–59	yes	10/02/20	NA	pos	NA	NA	ND	ND	ND	ND	MSFAC	A	Case 2	5	6–7
8	40–49	yes	11/02/20	5	neg	NA	NA	8.04/1.6	77.94	1/80	pos	MSFAC	C	Case 7	1	1
9	30–39	yes	11/02/20	6	pos	NA	NA	9.93/5	112.7	1/80	pos	MSFAC	F	Case 6	3	4
10	50–59	yes	13/02/20	23	pos	NA	NA	> 13/6.7	80.29	1/160	pos	MSFAC	D	Case 7	3	2–3
11	20–29	yes	14/02/20	21	pos	33	32	4.21/1	50.48	1/40	pos	MSFAC	E	Case 3	9	4
12	30–39	yes	14/02/20	11	ND	ND	ND	4.71/2.9	53.18	< 1/40	pos	MSFAC	F	Case 9	3	1–4
13	40–49	yes	15/02/20	18	ND	ND	ND	1.13/0.9	4.5	1/40	pos	MSFAC	G	Case 5	7	8
14	40–49	yes	16/02/20	18	pos	NA	NA	> 13/> 9	66.86	1/160	pos	Other facility	–	Case 8	5	1–5
15	40–49	yes	17/02/20	9	pos	37	33	ND	ND	ND	ND	Other facility	–	Case 14	1	1
16	40–49	yes	18/02/20	26	ND	ND	ND	3.2/1	18.95	1/40	pos	MSFAC	H	Case 11	4	4–6
17	50–59	yes	19/02/20	NA	pos	NA	NA	ND	ND	ND	ND	MSFAC	I	Case 5	11	2
18	50–59	yes	20/02/20	13	pos	NA	NA	11.3/2.1	15.91	1/80	pos	MSFAC	I	Case 17	1	2
19	20–29	yes	21/02/20	6	pos	NA	NA	4.42/1.8	91.23	1/40	pos	MSFAC	D	Case 10	8	9–10
20	40–49	yes	21/02/20	16	pos	NA	NA	1.78/2.7	1.5	1/40	pos	MSFAC	I	Case 17	2	2
21	30–39	yes	24/02/20	12	pos	37	34	12.8/ > 10	130	1/80	pos	MSFAC	J	Case 10	11	10
22	40–49	yes	25/02/20	20	pos	22	21	7.84/3.7	96.35	1/40	pos	MSFAC	K	Case 20	4	1–5
23	40–49	yes	25/02/20	2	ND	ND	ND	5.97/3.9	38.09	1/80	pos	MSFAC	L	Case 10	12	11
24	30–39	yes	10/03/20	5	ND	ND	ND	6.39/> 10	14.51	1/40	pos	MSFAC	M	Case 21	15	13
25	20–29	no	–	–	ND	ND	ND	1.76/7	24.09	< 1/40	pos	MSFAC	I	NA	–	–
26	20–29	no	–	–	ND	ND	ND	pos		1/40	pos	MSFAC	N	Case 2	–	–
27	30–34	no	–	–	ND	ND	ND	3.18/2.7	66.5	NA	pos	MSFAC	O	Case 10	–	–

*Ct* Cycle threshold (quantitative result of the RT-PCR)

*NA* Not available, *ND* Not done

*MSFAC* Military support facility of the airbase of Creil

The median age of confirmed cases was 40 (24 to 59), and the sex ratio was 15/12. Nineteen symptomatic cases (79%, 95% CI, 63–95) presented respiratory infections. In total, symptoms were fever ≥38 °C (*n* = 16, 67%), cough (*n* = 15, 63%), myalgia (*n* = 14, 58%), asthenia (*n* = 9, 38%), rhinorrhea (*n* = 8, 33%), headache (*n* = 8, 33%), odynophagia (*n* = 4, 17%), and diarrhea (*n* = 3, 13%). At that time, we did not systematically ask about ageusia/anosmia; only five patients declared them spontaneously. Four patients (17%) had dyspnea, two of whom were hospitalized in intensive care units at public hospitals. No healthcare workers of the military health facility fell sick.

### Chain of transmission

**Fig. 2:**
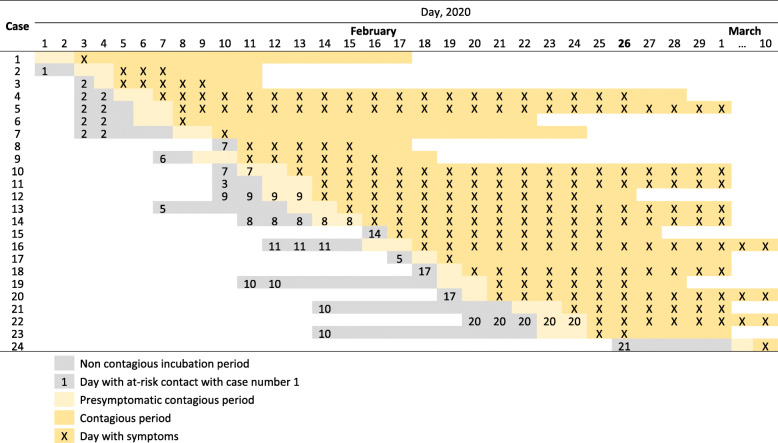
Inferred chain of transmission in MSFAC cluster, Creil, February–March 2020 (*n* = 24)

Based on contact tracing results and comparison of schedules for each case, it was possible to infer a chain of transmission (Fig. 2). The first infected service member (Case 2) developed the first symptoms on February 5. He had previous contact with a confirmed case in the civilian community of the city of Crépy-en-Valois in Oise, who had experienced symptom onset 2 days before (Case 1). Subsequently, four possible generations of cases occurred inside MSFAC, with a 4-day median incubation period ranging from 1 to 13 days, and a serial interval of 3 days (min = 0, max = 15). The reproduction rate during this outbreak was 1.36.

Viral sequencing was attempted on four samples of the cohort. Only one sample from Case 22, collected on March 2, allowed a near complete genome to be obtained, with an average coverage of 71,036X (EPI_ISL_415650). This genome was compared to the sequence obtained from a sample collected on February 21 from Case 7 (EPI_ISL_429968), and to sequence EPI_ISL_415649, obtained from a civilian from Crépy-en-Valois. All three sequences were highly similar. The genome from Case 22 only differed from the Case 7 sequence by a single nucleotide change (G28857A) resulting in a mutation in the N protein (R195K). The Case 7 sequence itself differed from the Crépy-en-Valois linked sequence only at two positions (T1666C, synonymous and C23520T, resulting in the A653V change in the spike). This data is compatible with the transmission network inferred from the epidemiological data (Fig. 2).

All cases from MSFAC worked in the same building, where the attack rate was 29% (24/83). We studied possible origins of contamination. At-risk types of contact were working in the same room (*n* = 9, 35%), using the same car (6, 23%), eating together (4, 15%), having a work relationship (4, 15%), belonging to the same household (2, 8%), and having a close-contact sports activity outdoors (1, 4%). Eventually, one swab from a shared object on Desk A (Table 1) was positive for SARS-CoV-2 among 17 environmental samples collected inside the building (Ct = 38), but the viral culture was negative.

### Countermeasures

In addition to the national preventive measures recommended by the Ministry of Health (physical distancing and individual hygiene), we put in place an action plan in collaboration with air base authorities. The MSFAC building was decontaminated with virucidal products. Within the MSFAC, only a small team dealt with current affairs while everyone else was quarantined at home with instructions to prevent other family members from being infected and told to report to the military health facility in case of symptoms. At that time, France was dealing with face mask shortages, and contingency capacity strategies were implemented so that face masks and bottles of alcohol-based hand sanitizer were systematically given to confirmed cases only. Transmission stopped immediately (no infection was detected after February 26).

MSFAC is a well-delimited facility housed inside a single building, but to limit any possibility of spread around it, work schedules were reorganized in all the other units on the air base to limit the number of people present on site and to keep several teams as backup in case new clusters were detected. This staff reduction also allowed physical distancing to be respected. Departments were separated in space and time to limit staff crossing paths, especially at lunchtime. Meetings were held by audio/video conference whenever possible. Public spaces (cinema, gyms) were closed, and exchanges and visits to other units were stopped.

## Discussion

This field investigation on patients infected with SARS-CoV-2 made it possible to observe the true reality of a SARS-CoV-2 epidemic and provided a good understanding of how to manage clusters or outbreaks. Such results also enable theoretical modeling to be validated or adjusted. Despite a lack of resources at that time (masks, testing), taking adequate and well-adapted measures stopped viral transmission in a few days. However, the results of this investigation should be confronted with those of other observational studies.

All cases in MSFAC were linked to each other within a single chain of transmission. The epidemiological investigation and viral genome sequencings were concordant and compatible with an introduction originating from a previous local circulation in Oise, from the Crépy-en-Valois cluster, which is described elsewhere by Fontanet et al. [12].

Airborne, droplet, and hand-to-hand transmissions were suspected, as 23 cases worked at the same place or together, and an environmental sample was positive for SARS-CoV-2 (despite the late collection date). Viral density in a confined area might have increased the reproductive rate, and viral inoculum at the time of infection could play a crucial role in the expression of the disease and the occurrence of severe cases (2/27, 7% in this cluster), [18].

Aside from ageusia and anosmia, which were not described at the start of the epidemic, clinical signs of SARS-CoV-2 infection are not specific, and COVID-19 mimicked co-circulating flu or colds. The first case identified (Case 7), who was not the index case but the starting point of this alert, was tested 14 days after the onset of symptoms, and 8 days after he deteriorated clinically, as he did not meet the epidemiological criteria of a possible case at that date. A lesson learned from this episode is that epidemiological case definitions are useful to standardize counting of cases everywhere, but they must not replace clinical diagnosis, especially during the emergence of new pathogens, because the infection and symptoms they cause are not well known and have not yet been described. When Case 7 was finally diagnosed, other primary, secondary, tertiary, and quaternary generations of cases had already been infected. The entire chain of transmission had to be identified backwards and forwards as early as possible to prevent an uncontrolled amplification of the outbreak [19, 20].

Deployment of molecular diagnostic tests was crucial. Due to a shortage of swabs and viral transport medium, our testing capability was limited (as was the case throughout France at that time). We defined the cluster area based on active case finding and contact tracing results and tested all symptomatic individuals within that perimeter. This testing strategy, based on medical evaluation of at-risk exposure, symptoms, and RT-PCR quantitative results, made it possible to limit false positives (and inappropriate lockdowns or closings) [21] and false negatives (we collected a second RT-PCR sample if any doubt existed). The effective use of targeted testing according to pre-test probability seems to be a key point in the successful management of outbreaks.

Regarding the possibility of missing asymptomatic cases, serology results confirmed the low percentage of patients who remained asymptomatic (13%), and contact tracing results did not identify any transmission from asymptomatic to symptomatic cases in this cluster. In a meta-analysis, Madewell et al. compared the secondary attack rate around symptomatic (18%) and asymptomatic COVID-19 cases (0.7%, *p* < 0.001) [22]. Nowadays, questions are being raised about the real role of asymptomatic infected people in the spread of the disease [23–27]. Among SARS-CoV-2 positive individuals, there is a confusion between those who are asymptomatic (= no symptoms at all), which is the case for 20% of COVID-19 patients, CI 95%, 17–25 according to the review of Buitrago-Garcia et al. [28], and those who are pre-symptomatic (= no symptoms at the time of sample collection for RT-PCR) [3, 29]. Indeed, Case 2 probably contaminated the five following cases during her pre-symptomatic stage, and 10 pre-symptomatic contaminations (about 40%) occurred in this cluster, highlighting the need for contact tracing and identifying, testing, quarantining and/or mask wearing for asymptomatic at-risk contact persons [30]. This is a major difference with SARS-CoV-1, where infectiousness started after symptom onset and was proportional to the clinical expression, making it easier to control [31].

Once the cluster area was defined, we placed the MSFAC staff under lockdown to slow down transmission within military settings during the investigation and contact tracing period. Ideally, increasing the use of digital tools and staff for timely contact tracing and early identification of the entire chain of transmission could reduce the duration of a lockdown [20, 32]. Six symptomatic cases (22% of the cases) did not go to the military health facility to be diagnosed and isolated, but no secondary cases occurred around them, probably due to the lockdown. Symptomatic cases that are not isolated are responsible for spreading the outbreak and underline the importance of raising awareness about COVID-19 symptoms within the population [33]. They also justify systematic mask wearing by at-risk populations [34].

Regarding the air base outside the cluster, since no significant viral circulation was identified, a lockdown was not required, but staff turnover, physical distancing, and hand hygiene measures were strongly implemented, without halting all activities.

All cases with a positive RT-PCR result and who underwent serological testing had a positive anti-SARS-CoV-2 serology with neutralizing antibodies, which confirmed a specific immunity against SARS-CoV-2, even if its duration and protective effect against further re-infection with variants are still unknown [10]. A longer follow-up of these patients would be necessary to conclusively establish that immunity.

## Conclusion

A rigorous investigation strategy based on systematic testing of at-risk symptomatic patients, with isolation of cases and at-risk contact persons, made it possible to quickly stop the spread of this COVID-19 cluster.

## Data Availability

The data sets generated and/or analyzed during the current study are not publicly available as they represent confidential medical information, but they are available from the corresponding author upon reasonable request. Viral sequencings are available online in the GISAID database (https://www.gisaid.org/). The anonymous individual identifiers are in the manuscript (= EPI_ISL_XXXX).
